# Expression of metalloproteinases (MMP-2 and MMP-9) in basal-cell carcinoma

**DOI:** 10.1007/s11033-016-4040-9

**Published:** 2016-07-12

**Authors:** Anna Goździalska, Anna Wojas-Pelc, Jagoda Drąg, Paweł Brzewski, Jerzy Jaśkiewicz, Maciej Pastuszczak

**Affiliations:** 1 Department of Health and Medical Sciences, Andrzej Frycz Modrzewski Krakow University, 1 G. Herlinga-Grudzińskiego St, 30-705 Krakow, Poland; 2 Department of Dermatology, Jagiellonian University Medical College, Krakow, Poland

**Keywords:** Basal-cell carcinoma, Matrix metalloproteinases, Type IV collagen, Molecular diagnosis, Invasiveness, Resection margin

## Abstract

The aim of this study was to compare the expressions of mRNA for metalloproteinases (MMP-2 and MMP-9) and type IV collagen in two different histological types of basal-cell carcinoma (BCCs; nodular and infiltrative) and in normal tissues from the tumor interface. The study included biopsy specimens of the skin involved with BCC and normal skin adjacent the lesion. The expressions of mRNA for MMP-2, MMP-9 and type IV collagen were determined by means of RT-PCR (Reverse transcription polymerase chain reaction). The level of type IV collagen mRNA in nodular and infiltrative BCCs turned out to be significantly lower, and the expressions of MMP-2 and MMP-9 mRNA significantly higher than in normal tissues adjacent to these tumors. The expression of mRNA for MMP-9 but not for MMP-2 was significantly higher in infiltrative BCCs than in the nodular BCCs. In turn, normal tissues adjacent to nodular BCCs showed significantly higher levels of mRNA for MMP-2 and significantly lower levels of type IV collagen mRNA than the normal tissues from the interface of infiltrative BCCs. The findings suggest that MMP-2 and MMP-9 could be used as prognostic factors of BCCs.

## Introduction

Cancers constitute a significant problem of modern medicine [1], being the second leading cause of mortality worldwide [2, 3]. Despite extensive research, the pathomechanism of carcinogenesis is still not completely understood. Non-melanoma skin cancers (NMSCs) are the most frequent human malignancies, characterized by constantly increasing incidence [4–6]. Basal-cell carcinoma (BCC), especially its nodular and infiltrative forms, represent a considerable fraction of all NMSCs (75–80 %) [7–9]. A dramatic increase in the incidence of BCC has been recently observed in Europe, United States and Australia, especially among Caucasians [10, 11]. Although BCC is typically diagnosed at older age, mean age at diagnosis still decreases, and this malignancy can be sporadically found in teenagers [6].

BCC is characterized by a slow infiltrative growth resulting in destruction of surrounding structures. Most BCCs are not invasive, rarely form metastases or lead to mortality [9, 12]. However, delayed diagnosis and resultant local progression of the tumor may lead to involvement of surrounding tissues [13, 14] and serious anatomical deformations, especially in the case of face [4, 15]. This may exert significant detrimental effect on the quality of life of BCC patients [16, 17].

Metalloproteinases 2 and 9 (MMP-2 and MMP-9), enzymes from the gelatinase family, and their inhibitors play important role in the progression of BCC [18–20]. Due to degradation of type IV collagen by MMP, cancer cells can migrate outside the tumor and form distant metastases [21]. A key role in this process is ascribed to MMP-9, which acts as a promotor of tumor invasion [22, 23]. An increase in the expression of MMP-9 was shown to correlate with clinical stage of BCC and more aggressive phenotype of this cancer [21, 24, 25]. Furthermore, MMP-9 plays an important role in the process of neoangiogenesis, being involved in the proliferation of endothelial cells and activation of pro-angiogenic factors [20, 24–27].

Correct diagnosis constitutes a crucial component of BCC management. Diagnosed at early stages, this cancer is fully curable. Most BCCs of the skin can be readily diagnosed on physical examination [6, 28]. Macroscopic evaluation of the tumor is usually sufficient for establishing correct diagnosis, and histopathological analysis allows to identify the type of tumor growth [9]. Furthermore, microscopic examination of the biopsy specimen is crucial for therapeutic decisions, namely selection of optimal technique for surgical resection. Unfortunately, the resections of BCC are suboptimal in many cases which is reflected by high rates of local recurrences [29]. This typically results from the problems with correct identification of resection margin.

Progress in molecular biology has revolutionized approach to diagnosis and treatment of cancers [30, 31]. Molecular examination of the biopsy specimens can be helpful in determining the stage of tumor and appropriate identification of resection margins. Consequently, we decided to verify if molecular techniques can be helpful in the diagnosis and management of BCC. Therefore, the aim of this study was to compare the expressions of mRNA for metalloproteinases (MMP-2 and MMP-9) and type IV collagen in two different histological types of BCC (nodular and infiltrative) and in normal tissues from the tumor interface.

## Materials and methods

### Patients

The protocol of the study was approved by the Local Bioethics Committee at the Jagiellonian University in Krakow (decision no. KBET/37/B/2009).

The study included biopsy specimens of the skin involved with BCC, as well as the biopsy specimens from normal skin adjacent the lesion. The samples from the tumors and normal skin were obtained from the same patients (n = 70, 28 women and 42 men), during surgical resections of BCCs, performed at the Clinical Department of Dermatology, University Hospital in Krakow. All of patients are Caucasian. 35 patients (14 women and 21 men) presented with infiltrative BCCs, and another 35 (14 women and 21 men) with nodular BCCs. Mean age of the patients was 68 ± 10.6 years for women and 67 ± 10.8 years for men with infiltrative BCCs and 74 ± 6.7 years for women and 76 ± 6.5 years for men with nodular BCCs.

### Methods

The biopsy specimens from BCCs and normal adjacent skin were used to determine the expressions of mRNA for MMP-2 (*MMP*-*2*), MMP-9 (*MMP*-*9*) and type IV collagen (*COL4A4*) genes, as well as for beta-actin gene (*ACTB*), used as a reporter gene. 30-mg samples were obtained from each specimen, previously fixed in a RNAlater RNA Stabilization Reagent (Qiagen, Germany), and pulverized in liquid nitrogen. Total RNA was isolated at 4 °C in a DNA/RNA UV-Cleaner UVC/T-AR chamber (Biosan, Latvia), using a modified method described by Chomczynski and Sacchi [32]. Concentration and purity of the extracted RNA were determined spectrophotometrically.

The samples for reverse transcription were prepared in the DNA/RNA UV-Cleaner UVC/T-AR chamber (Biosan, Latvia). The reaction was conducted with an aid of a Revert Aid H Minus First Strand cDNA Synthesis Kit (Fermentas, Lithuania). The resultant cDNA templates were subjected to PCR with an Opti Taq hot start polymerase (Eur_x,_ Poland) and a set of specific primers (*MMP*-*2*: F5′-CACTTTCCTGGGCAACAAAT-3′ and R5′-CTCCTCAATGCCCTTGATGT-3′ at 10 μM; *MMP*-*9*: F5′-TCCCTGGAGACCTGAGAACC-3′ and R5′-GTCGTCGGTGTCGTAGTTGG-3′ at 10 μM; *COL4A4*: F5′-CCCCTCAGGACCAGGGTGCAA-3′ and R5′-AGGGGCGGATCGCCTCTTCCA-3′ at 4 μM; *ACTB*: F5′-GGACTTCGAGCAAGAGATGG-3′ and R5′-AGCACTGTGTTGGCGTACAG-3′ at 6 μM). All the primers were synthesized at DNA Gdansk (Poland). The resultant amplicons of MMP-2 (271 kbp), MMP-9 (659 kbp), COL4A4 (482 kbp) and ACTB (234 kbp) were separated by electrophoresis in a 1.5 % agarose gel with ethidium bromide, using a horizontal electrophoresis system (Kucharczyk, Poland). The electrophoretograms were photographed with an aid of a digital camera (Olympus, USA), UV transilluminator (Vilber Lourmat, France) and PolyDoc system for electrophoretic gel documentation and analysis. The digital recordings of the electrophoretograms were subjected to densitometric analysis with Quantity One 4.2.1 software (Bio-Rad, USA). The density of the RT PCR products was considered as an equivalent of MMP-2, MMP-9, type IV collagen and beta-actin mRNA expressions.

### Statistical analysis

Statistical analysis was conducted with a SPSS 18 package (IBM, USA). Continuous variables were examined with Kolmogorov–Smirnov test for normality of their distributions, and Student *t* test was used for intergroup comparisons. The results of the two tests were considered significant at p ≤ 0.05.

## Results

**Fig. 1:**
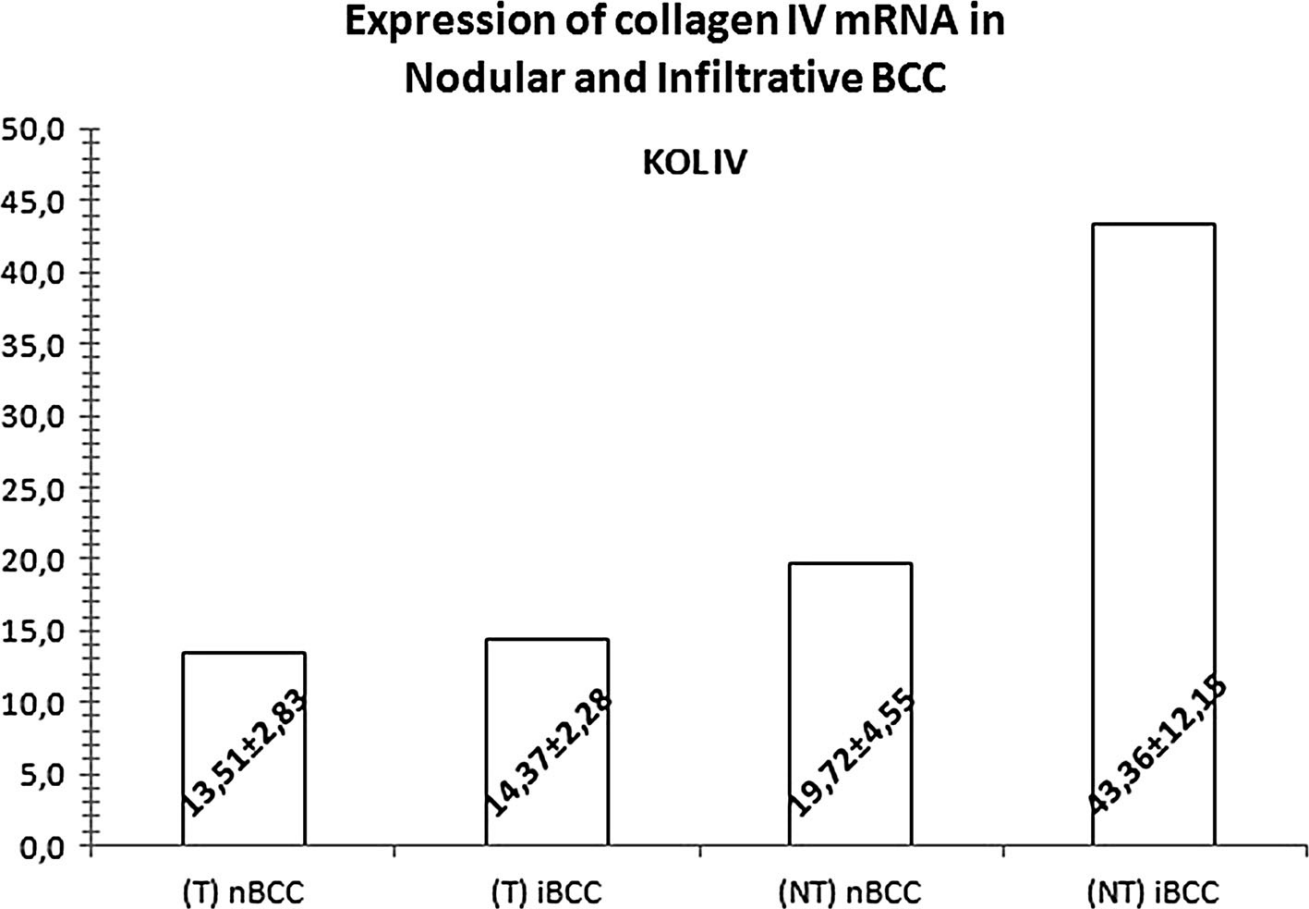
Mean (±SD) expressions of mRNA for type IV collagen in the tissues (T) of nodular (nBCC) and infiltrative (iBCC) basal-cell carcinoma and in normal tissues adjacent to the tumors (NT)

**Fig. 2:**
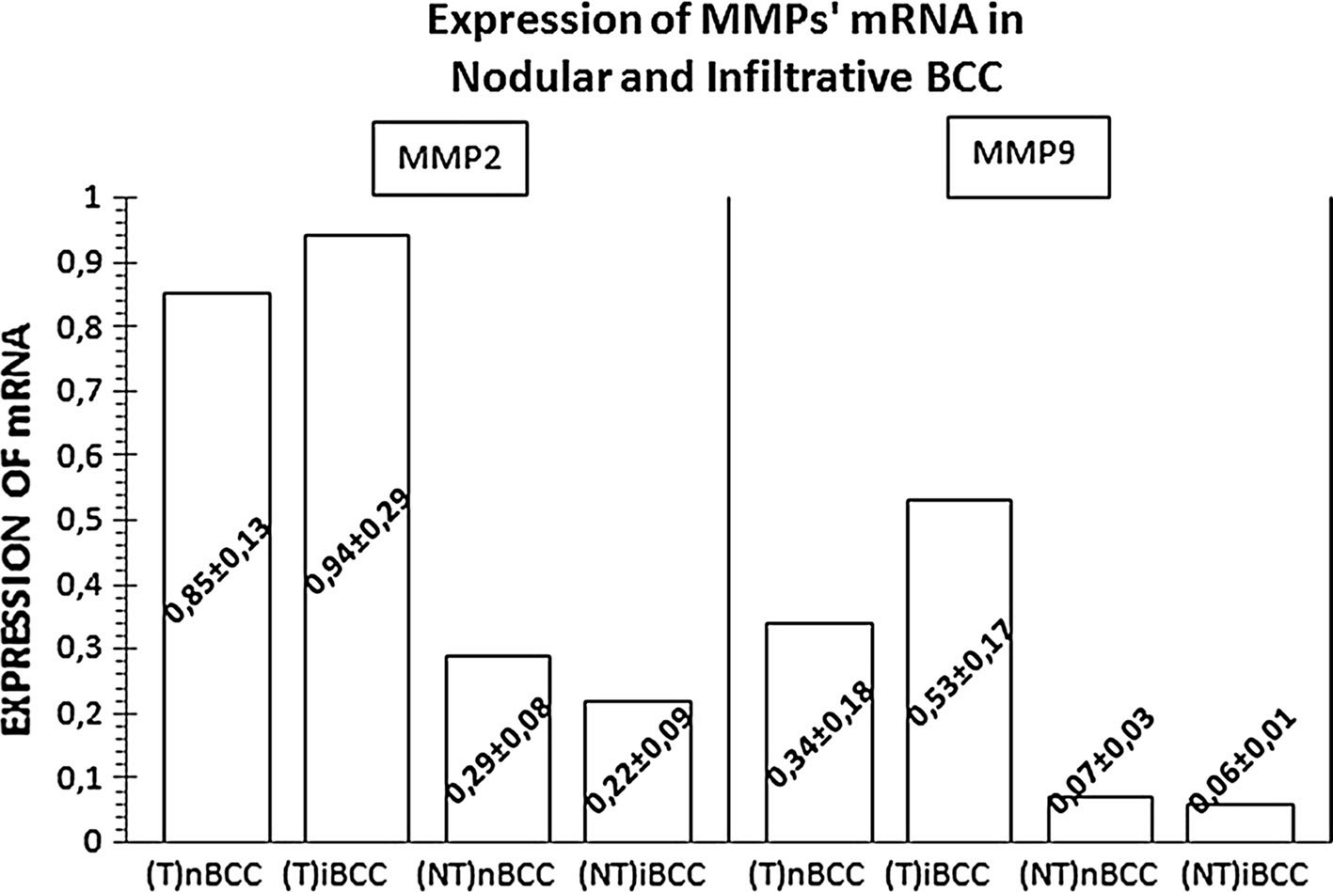
Mean (±SD) expressions of mRNA for matrix metalloproteinases 2 (MMP2) and 9 (MMP9) in the tissues (T) of nodular (nBCC) and infiltrative (iBCC) basal-cell carcinoma and in normal tissues adjacent to the tumors (NT)

The level of type IV collagen mRNA in nodular BCCs turned out to be significantly lower (by 31 %), and the expressions of MMP-2 and MMP-9 mRNA significantly higher (by 293 and 486 %, respectively) than in normal tissues adjacent to these tumors (p < 0.001 for all the comparisons). Also in the case of infiltrative BCCs, the expression of type IV collagen mRNA was shown to be significantly lower (by 67 %) and the levels of MMP-2 and MMP-9 mRNA significantly higher (by 427 and 883 %, respectively) than in the adjacent normal tissues (p < 0.001 for all the comparisons; Figs. 1, 2).

The expression of mRNA for MMP-9 (56 % difference, p < 0.001), but not for MMP-2 (11 % difference, p = 0.097), turned out to be significantly higher in infiltrative BCCs than in the nodular BCCs. The two tumor types did not differ significantly in terms of their type IV collagen mRNA expressions (p = 0.166). In turn, normal tissues adjacent to nodular BCCs showed significantly higher levels of mRNA for MMP-2 (32 % difference, p = 0.001), but not MMP-9 (17 % difference, p = 0.209), and significantly lower levels of type IV collagen mRNA (17 % difference, p < 0.001) than the normal tissues from the interface of infiltrative BCCs (Figs. 1, 2).

In group of nodular BCC, between women and men, the expression of mRNA for MMP-2, MMP-9 and type IV collagen did not differ significantly (Table 1). In group of infiltrative BCC, only expression of type IV collagen was significantly higher in men as compared to women (Table 2).

**Table 1:** Changes in mRNA expression of MMP2, MMP9 and kol IV in nodular BCCs, depending on gender

	MMP2 T	MMP2 NT	MMP9 T	MMP9 NT	KOL IV T	KOL IV NT
Women	0.83 ± 0.17	0.26 ± 0.08	0.32 ± 0.1	0.07 ± 0.03	13.58 ± 2.16	18.94 ± 2.86
Men	0.87 ± 0.1	0.31 ± 0.08	0.35 ± 0.2	0.07 ± 0.03	13.47 ± 3.24	20.24 ± 5.4

**Table 2:** Changes in mRNA expression of MMP2, MMP9 and kol IV in infiltrative BCCs, depending on gender

	MMP2 T	MMP2 NT	MMP9 T	MMP9 NT	KOL IV T	KOL IV NT
Women	1.03 ± 0.37	0.25 ± 0.11	0.53 ± 0.21	0.06 ± 0.01	15.06 ± 2.52	37.01 ± 7.45*
Men	0.89 ± 0.22	0.20 ± 0.06	0.54 ± 0.13	0.06 ± 0.01	13.91 ± 2	47.59 ± 12.9*

*Statistically significant differences

## Discussion

We analyzed the expressions of mRNA transcripts for MMP-2, MMP-9 and type IV collagen in nodular and infiltrative BCCs. Quantitative analysis of mature mRNA transcripts enabled us to appropriately asses the expression of these proteins, as their biosynthesis is mostly regulated at a transcriptional and post-transcriptional level. Examination of mature mRNA transcripts (possible due to application of appropriate primers) is suitable for the evaluation of gene expression after the post-transcriptional modification.

### Expression of mRNA for MMP-2, MMP-9 and type IV collagen

We showed that the expressions of mRNA for MMP-2 and MMP-9 in nodular and infiltrative BCCs were significantly higher than in normal tissues adjacent to these tumors; this observation supports existing evidence on the involvement of metalloproteinases in carcinogenesis. MMP-2 and MMP-9 catalyze proteolysis of type IV collagen, and thus participate in the destruction of the basement membrane barrier. Due to presence of specific catalytic domain, MMP-2 and MMP-9 can degrade virtually all components of this barrier. Cancer cells can release metalloproteinases or stimulate synthesis thereof in normal tissues, e.g. via the secretion of extracellular matrix metalloproteinase inducer (EMMPRIM) [33, 34]. Moreover, metalloproteinases may be synthesized by the transformed cells themselves [35]. Enhanced proteolysis of extracellular matrix (ECM) and resultant degradation of type IV collagen, the main component of the basement membrane, enable growth of a tumor and migration of cancer cells to blood vessels, and then to distant tissues and organs [6].

Carcinogenesis is associated with overexpression of the metalloproteinase genes and increase in the enzymatic activity of their products. The overexpression is associated with regulation of genes at a transcriptional level, involving such transcription factors as AP-1 and AP-2. Cancer tissues were demonstrated to express latent forms of metalloproteinases and show decreased expression of metalloproteinase inhibitors, such as tissue inhibitors of metalloproteinases (TIMPs) and alpha-2-macroglobulin [36].

Both metalloproteinases (MMP-2and MMP-9) and type IV collagen are involved in angiogenesis, which is vital for tumor growth and invasion [37, 38]. Enhanced expression of mRNA for metalloproteinases leads to excessive degradation of ECM [39]. Under physiological conditions, the degradation of ECM is tightly controlled due to regulation of expression and activity of proteolytic enzymes [35]. However, this control mechanisms are disrupted under pathological conditions, e.g. in the BCC tissues [9]. The abovementioned changes in the expressions of MMP-2 and MMP-9 lead to structural alterations of the basement membrane and ECM [40]; as a result, endothelial cells can freely migrate inside the tumor and form new blood vessels necessary for its further growth [41, 42].

### Nodular versus infiltrative BCCs

The expression of mRNA for MMP-2 in nodular BCCs turned out to be lower than in the infiltrative BCCs. This points to greater invasiveness of the infiltrative BCC; catalyzing proteolysis of the basement membrane, metalloproteinases enable cancer cells to infiltrate to adjacent tissues. In contrast, the expression of MMP-2 mRNA in normal cells obtained from the margin of nodular BCCs was stronger than in the normal cells located at an interface of the infiltrative BCCs. This likely reflected silencing of the mRNA expression in normal cells adjacent to infiltrative BCCs. In turn, the overexpression of MMP-2 mRNA in tissues surrounding nodular BCCs may a kind of counterbalance effect: cancer cells from the collagen-entrapped tumor may stimulate the synthesis of MMP-2 in normal adjacent cells, e.g. acting via the EMMPRIM protein [34].

We showed that nodular BCCs were characterized by significantly lower levels of MMP-9 mRNA than the infiltrative tumors. This observation is consistent with the results of many previous studies in which the invasiveness of infiltrative BCCs was shown to be determined by their elevated expression of MMP-9 [43–46].

Nodular BCCs were characterized by slightly lower levels of type IV collagen mRNA than the infiltrative BCCs [47, 48]. In turn, the expression of type IV collagen mRNA in normal tissues from the interface of nodular BCCs turned out to be significantly lower than in the normal tissues surrounding infiltrative BCCs. Perhaps normal cells adjacent to infiltrative BCCs show stronger expression of mRNA for type IV collagen in order to prevent their infiltration by cancer cells. Infiltrative BCCs were shown to be more invasive than the nodular BCCs, isolated from surrounding tissues by a capsule composed of type I and III collagen [8].

ECM undergoes constant remodeling catalyzed by proteolytic enzymes, among them metalloproteinases. A number of previous studies showed an increase in the expressions of MMP-2 and MMP-9 within various malignancies, and these enzymes were proposed as potential cancer markers [25, 44, 49–52]. Our findings are consistent with the data published by Vempati et al. [46], Zlatarova et al. [25], Fu et al. [50], El-Khalawany and Abou-Bakr [24] and Vanjaka-Rogosic et al. [53], who showed that the expression of MMP-9 may constitute a molecular marker of processes taking place within the BCCs. However, the evidence in this matter is inconclusive. For example, Ciurea et al. [54] claimed that MMP-9 does not accurately distinguish between the BCC and normal tissue.

### Molecular markers of carcinogenesis

The proteolysis of ECM results from the activity of various MMPs synthesized by a number of normal and neoplastic cells [55]. The degradation of extracellular matrix is also supported by many hormones, cytokines and growth factors that induce synthesis of proteolytic enzymes and their inhibitors [39].

Varani et al. [45] showed that while most cancer tissues express active forms of MMP-2 and MMP-9, the latent forms of these enzymes can be predominantly found in the normal tissues. According to Orimoto et al. [52], MMP-2 is a very accurate marker distinguishing between BCCs and surrounding normal tissues. In contrast, Chen et al. [49] demonstrated the inhibition of MMP-2 expression in BCC and put the diagnostic value of this marker into question. These discrepancies substantiate further research on the problem in question.

## Conclusion

Cancer cells synthesize an array of factors that perpetuate their proliferation and migration, and induce pathological angiogenesis. At least some of these factors can be used as markers of invasiveness, used to distinguish between pathological and normal tissue at the molecular level. It is of vital importance, as microscopic identification of the tumor margin is often inaccurate, resulting in suboptimal resection of BCC. Another potential application of novel molecular markers is detection of carcinogenesis at early preclinical stages [56]. Our hereby presented findings suggest that MMP-2 and MMP-9 could be used as prognostic factors of BCCs.

## References

[1] Kułakowski A, Skowrońska-Gardas A (2007). Onkologia.

[2] Kumar V, Cotran R, Robbins S (2005). Patologia.

[3] Stachura J, Domagała W (2008). Patologia znaczy słowo o chorobie.

[4] Chicheł A, Skowronek J (2005). Contemporary management of skin cancer–dermatology, surgery or radiotherapy?. Contemp Oncol.

[5] Cumberland L, Dana A, Liegeois N (2009). Mohs micrographic surgery for the management of nonmelanoma skin cancers. Facial Plast Surg Clin North Am.

[6] Pabiańczyk R, Cieślik K, Tuleja T (2011). Management methods of basal cell carcinoma. Chir Pol.

[7] Baran E (2008). Nowotwory skóry Klinika, patologia, leczenie.

[8] Bolognia J, Jorizzo J, Rapini R (2012). Dermatology.

[9] Cigna E, Tarallo M, Maruccia M, Sorvillo V, Pollastrini A, Scuderi N (2011). Basal cell carcinoma: 10 years of experience. J Skin Cancer.

[10] Staples MP, Elwood M, Burton RC, Williams JL, Marks R, Giles GG (2006). Non-melanoma skin cancer in Australia: the 2002 national survey and trends since 1985. Med J Aust.

[11] Weedon D (2010). Weedon’s skin pathology.

[12] Błaszczyk-Kostanecka M, Wolska H (2009). Dermatologia w praktyce.

[13] Rigel DS (2008). Cutaneous ultraviolet exposure and its relationship to the development of skin cancer. J Am Acad Dermatol.

[14] Saldanha G, Fletcher A, Slater DN (2003). Basal cell carcinoma: a dermatopathological and molecular biological update. Br J Dermatol.

[15] Daniel L, Leśniewski-Kmak K (2005). Treatment of basal cell epithelioma which has been growing for many years: a case report. Contemp Oncol.

[16] Gawkrodger D, Ardern-Jones M (2012). Dermatology: an illustrated colour text.

[17] Ghanadan A, Abbasi A, Rabet M, Abdollahi P, Abbasi M (2014). Characteristics of mixed type basal cell carcinoma in comparison to other BCC subtypes. Indian J Dermatol.

[18] Chen W, Fu X, Ge S, Sun T, Sheng Z (2007). Differential expression of matrix metalloproteinases and tissue-derived inhibitors of metalloproteinase in fetal and adult skins. Int J Biochem Cell Biol.

[19] Hernandez-Perez M, El-hajahmad M, Massaro J, Mahalingam M (2012). Expression of gelatinases (MMP-2, MMP-9) and gelatinase activator (MMP-14) in actinic keratosis and in in situ and invasive squamous cell carcinoma. Am J Dermatopathol.

[20] Monhian N, Jewett BS, Baker SR, Varani J (2005). Matrix metalloproteinase expression in normal skin associated with basal cell carcinoma and in distal skin from the same patients. Arch Facial Plast Surg.

[21] Roh MR, Zheng Z, Kim HS, Kwon JE, Jeung HC, Rha SY (2012). Differential expression patterns of MMPs and their role in the invasion of epithelial premalignant tumors and invasive cutaneous squamous cell carcinoma. Exp Mol Pathol.

[22] Kerkela E, Saarialho-Kere U (2003). Matrix metalloproteinases in tumor progression: focus on basal and squamous cell skin cancer. Exp Dermatol.

[23] Poswar FO, Fraga CA, Farias LC, Feltenberger JD, Cruz VP, Santos SH (2013). Immunohistochemical analysis of TIMP-3 and MMP-9 in actinic keratosis, squamous cell carcinoma of the skin, and basal cell carcinoma. Pathol Res Pract.

[24] El-Khalawany MA, Abou-Bakr AA (2013). Role of cyclooxygenase-2, ezrin and matrix metalloproteinase-9 as predictive markers for recurrence of basal cell carcinoma. J Cancer Res Ther.

[25] Zlatarova ZI, Softova EB, Dokova KG, Messmer EM (2012). Expression of matrix metalloproteinase-1, -9, -13, and tissue inhibitor of metalloproteinases-1 in basal cell carcinomas of the eyelid. Graefes Arch Clin Exp Ophthalmol.

[26] Chang C, Werb Z (2001). The many faces of metalloproteases: cell growth, invasion, angiogenesis and metastasis. Trends Cell Biol.

[27] Łukaszewicz-Zając M, Mroczko B, Szmitkowski M (2009). The signifi cance of metalloproteinases and their inhibitors in gastric cancer. Postep Hig Med Dosw.

[28] Sterry W, Paus R, Burgdorf W (2009). Dermatologia.

[29] Bartos V, Pokorny D, Zacharova O, Haluska P, Doboszova J, Kullova M (2011). Recurrent basal cell carcinoma: a clinicopathological study and evaluation of histomorphological findings in primary and recurrent lesions. Acta Dermatovenerol Alp Pannonica Adriat.

[30] Fitzpatrick TB (1988). The validity and practicality of sun-reactive skin types I through VI. Arch Dermatol.

[31] Włodarkiewicz A, Sobjanek M, Michajłowski I, Nałęcz D, Niekra M, Michajłowski D (2012). Molecular strategies in the treatment of skin cancers. Przegl Dermatol.

[32] Chomczynski P, Sacchi N (1987). Single-step method of RNA isolation by acid guanidinium thiocyanate-phenol-chloroform extraction. Anal Biochem.

[33] Caudroy S, Polette M, Nawrocki-Raby B, Cao J, Toole BP, Zucker S (2002). EMMPRIN-mediated MMP regulation in tumor and endothelial cells. Clin Exp Metastasis.

[34] Davidson B, Goldberg I, Berner A, Kristensen GB, Reich R (2003). EMMPRIN (extracellular matrix metalloproteinase inducer) is a novel marker of poor outcome in serous ovarian carcinoma. Clin Exp Metastasis.

[35] Nagase H, Visse R, Murphy G (2006). Structure and function of matrix metalloproteinases and TIMPs. Cardiovasc Res.

[36] Nagase H (1997). Activation mechanisms of matrix metalloproteinases. Biol Chem.

[37] Bogaczewicz J, Dudek W, Zubilewicz T, Wroński J, Przywara S, Chodorowska G (2006). The role of matrix metalloproteinases and their tissue inhibitors in angiogenesis. Pol Merk Lek.

[38] Nabeshima K, Inoue T, Shimao Y, Sameshima T (2002). Matrix metalloproteinases in tumor invasion: role for cell migration. Pathol Int.

[39] Nagase H, Woessner JF (1999). Matrix metalloproteinases. J Biol Chem.

[40] Dumas V, Kanitakis J, Charvat S, Euvrard S, Faure M, Claudy A (1999). Expression of basement membrane antigens and matrix metalloproteinases 2 and 9 in cutaneous basal and squamous cell carcinomas. Anticancer Res.

[41] Łapka A, Drąg J, Goździalska A, Jaśkiewicz J (2008). Matrix metalloproteinases in gliomas. Postep Psychiatrii Neurol.

[42] Śliwowska I, Kopczyński Z (2005). Matrix metalloproteinases–biochemical characteristics and clinical value determination in breast cancer patients. Contemp Oncol.

[43] Emara M, Woźniak M (1999). Role of metalloproteinases in cancer cell invasiveness. Diagn Lab.

[44] O’Grady A, Dunne C, O’Kelly P, Murphy GM, Leader M, Kay E (2007). Differential expression of matrix metalloproteinase (MMP)-2, MMP-9 and tissue inhibitor of metalloproteinase (TIMP)-1 and TIMP-2 in non-melanoma skin cancer: implications for tumour progression. Histopathology.

[45] Varani J, Hattori Y, Chi Y, Schmidt T, Perone P, Zeigler ME (2000). Collagenolytic and gelatinolytic matrix metalloproteinases and their inhibitors in basal cell carcinoma of skin: comparison with normal skin. Br J Cancer.

[46] Vempati P, Karagiannis ED, Popel AS (2007). A biochemical model of matrix metalloproteinase 9 activation and inhibition. J Biol Chem.

[47] Quatresooz P, Martalo O, Pierard GE (2003). Differential expression of alpha1 (IV) and alpha5 (IV) collagen chains in basal-cell carcinoma. J Cutan Pathol.

[48] Rasmussen HB, Teisner B, Andersen JA, Brandrup F, Purkis T, Leigh I (1991). Immunohistochemical studies on the localization of fetal antigen 2 (FA2), laminin, and collagen type 4 in basal cell carcinoma. J Cutan Pathol.

[49] Chen GS, Lu MP, Wu MT (2006). Differential expression of matrix metalloproteinase-2 by fibroblasts in co-cultures with keratinocytes, basal cell carcinoma and melanoma. J Dermatol.

[50] Fu XR, Zhang C, Chen CY, Zhang L, Wang LX, Wan BH (2012). Expression and significance of matrix metalloproteinase and tissue inhibitor of matrix metalloproteinase in non-melanoma skin cancer. Zhonghua Zhong Liu Za Zhi.

[51] Kwiatkowski P, Godlewski J, Śliwińska-Jewsiewicka A, Kmieć Z (2008). The role of matrix metalloproteinases in tumour invasion. Pol Ann Med.

[52] Orimoto AM, Neto CF, Pimentel ER, Sanches JA, Sotto MN, Akaishi E (2008). High numbers of human skin cancers express MMP2 and several integrin genes. J Cutan Pathol.

[53] Vanjaka-Rogosic L, Puizina-Ivic N, Miric L, Rogosic V, Kuzmic-Prusac I, Babic MS (2014). Matrix metalloproteinases and E-cadherin immunoreactivity in different basal cell carcinoma histological types. Acta Histochem.

[54] Ciurea ME, Cernea D, Georgescu CC, Cotoi OS, Patrascu V, Parvanescu H (2013). Expression of CXCR4, MMP-13 and beta-catenin in different histological subtypes of facial basal cell carcinoma. Rom J Morphol Embryol.

[55] Edwards D, Appelt K, Totowa N, Clendennin N (2001). Matrix Metalloproteinases Inhibitors in Cancer Therapy. The tissue inhibitors of metalloproteinases (TIMPs).

[56] Kowalski M, Walczak A, Majsterek I (2008). Matrix metalloproteinases (MMPs): modern molecular markers of open-angle glaucoma diagnosis and therapy. Postep Hig Med Dosw.

